# Calciphylaxis following acute renal injury: a case and literature review

**DOI:** 10.1186/s40064-016-2740-1

**Published:** 2016-07-11

**Authors:** Tomoko Oda, Yu Sawada, Takashi Yamaguchi, Shun Ohmori, Daisuke Omoto, Sanehito Haruyama, Manabu Yoshioka, Etsuko Okada, Motonobu Nakamura

**Affiliations:** Department of Dermatology, University of Occupational and Environmental Health, 1-1 Iseigaoka, Yahatanishi-ku, Kitakyushu, 807-8555 Japan

**Keywords:** Calciphylaxis, Acute renal injury, Literature review

## Abstract

**Background:**

Calciphylaxis following acute renal failure is rare.

**Findings:**

We report A 57-year-old male with an acute renal failure associated with necrotizing fasciitis. We also review the cases of calciphylaxis due to acute renal disorder further.

**Conclusions:**

It should be kept in mind that calciphylaxis is observed in patient with not only chronic renal disease but also acute renal failure.

## Background

Calciphylaxis is a disease of cutaneous vessel calcification-induced skin ulceration (Hayashi 2013). Although the detail mechanism remains unclear, various diseases, such as a secondary hyperparathyroidism associated with chronic renal failure, are known to trigger calciphylaxis (Hayashi 2013). However, some calciphylaxis cases have also been reported during hemodialysis for acute renal failure (Honda et al. 2015; Chavel et al. 2004). Herein, we report a case of calciphylaxis caused following acute renal failure. We also review the cases of calciphylaxis for hemodialysis due to acute renal disorder further.

## Case report

**Fig. 1:**
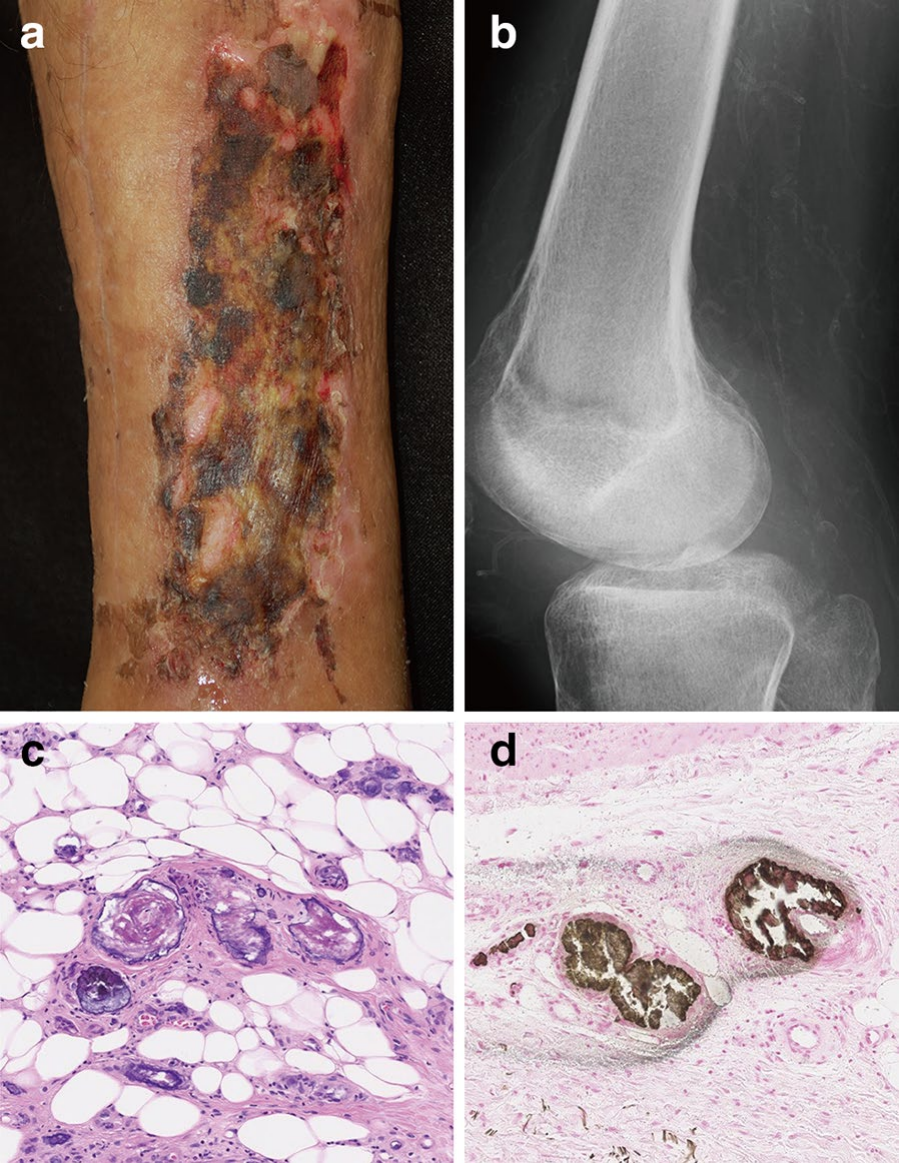
Clinical manifestation and laboratory examination. **a** Physical examination showing a skin ulcer covered with *black-yellowish* necrotic tissue on his lower leg. **b** Radiography demonstrated calcified vessels in both legs. **c** Hematoxylin and eosin stains showing thrombosis of vessels in skin biopsy specimen. **d** Von Kossa staining showing with calcium depositions in vessel walls

A 57-year-old male with an acute renal failure associated with necrotizing fasciitis underwent hemodialysis. He had no history of diabetes mellitus or heavy smoking. Six weeks after the hemodialysis, he noticed painful skin ulcers on both of his legs, which gradually enlarged without any rubbing or other outer physical stimuli. Physical examination revealed a skin ulcer covered with black-yellowish necrotic tissue (Fig. 1a). Dorsalis pedis pulse was palpable. Radiography demonstrated calcified vessels in both legs (Fig. 1b). A skin biopsy taken from his leg demonstrated a thrombosis of vessels (Fig. 1c) with calcium depositions (Fig. 1d). Biochemical profiles showed that hyperphosphatemia (13.4 mg/dl, normal <4.6 mg/dl) with hyperparathyroidism (intact PTH level 85 pg/ml, normal <65 pg/ml). Serum level of corrected calcium was 9.0 mg/dl (normal >8.8 mg/dl). Based on these examinations, we suspected that his skin ulcer was caused by calciphylaxis due to hyperparathyroidism associated with acute renal failure or hemodialysis. After a sodium thiosulfate administration, his skin eruption and pain gradually improved.

## Discussion

Although almost all cases with calciphylaxis have been described in patients with end-stage renal disease on hemodialysis, this patient exhibited calciphylaxis following hemodialysis due to acute renal failure. To clarify the detail characteristics, we review the cases of calciphylaxis associated with acute renal failure. There have been three reported cases including our case (Table 1) (Honda et al. 2015; Chavel et al. 2004). All cases underwent hemodialysis. The average duration of hemodialysis before the initial appearance of a skin ulcer was approximately 5 weeks, which is relatively short time compared to the cases of hemodialysis for chronic renal failure. It has been known that several cases also developed hyperparathyroidism after acute renal failure without hemodialysis (Bitran 1976) because of skeletal resistance to parathyroid hormone (Massry et al. 1974). Therefore, it was speculated that the acute disturbance of homeostasis due to acute renal failure or hemodialysis might also contribute to the development of calciphylaxis due to secondary hyperparathyroidism.

**Table 1:** Case reports of calciphylaxis due to acute renal failure

Author	Sex	Age	Hemodialysis	Hemodialysis duration	Treatment for calciphylaxis	Therapeutic response
Honda et al.	Female	47	+	1 month	Sodium thiosulfate Skin transplantation	Good
Chavel et al.	Male	47	+	4 weeks	Topical skin care	Poor
Our case	Male	57	+	6 weeks	Topical skin care Sodium thiosulfate	Moderate

## Conclusions

It should be kept in mind that calciphylaxis is observed in patient with not only chronic renal disease but also acute renal failure. Because of limited number of cases, further investigation is necessary to clarify the detail mechanism of calciphylaxis due to acute renal failure.
